# Identification and Functional Analysis of MicroRNAs and Their Targets in *Platanus acerifolia* under Lead (Pb) Stress

**DOI:** 10.3390/ijms16047098

**Published:** 2015-03-30

**Authors:** Yuanlong Wang, Zhenli Zhao, Minjie Deng, Rongning Liu, Suyan Niu, Guoqiang Fan

**Affiliations:** 1 Institute of Paulownia, Henan Agricultural University, 95 Wenhua Road, Jinshui District, Zhengzhou 450002, China; E-Mails: xiaoyunxia2012@126.com (Y.W.); zhaozhl2006@126.com (Z.Z.); dengmj1980@126.com (M.D.); lrn0501@163.com (R.L.); suyanniu_happy@126.com (S.N.); 2 Department of Landscape Architecture, Henan Vocational College of Agriculture, Zhengzhou 451450, China

**Keywords:** *Platanus acerifolia*, Pb stress, MicroRNA, degradome sequencing

## Abstract

MicroRNAs (miRNAs) play important regulatory roles in development and stress responses in plants. Lead (Pb) is a non-essential element that is highly toxic to living organisms. *Platanus acerifolia* is grown as a street tree in cities throughout temperate regions for its importance in improving the urban ecological environment. MiRNAs that respond to abiotic stresses have been identified in plants; however, until now, the influence of Pb stress on *P. acerifolia* miRNAs has not been reported. To identify miRNAs and predict their target genes under Pb stress, two small RNA and two degradome libraries were constructed from Pb-treated and Pb-free leaves of *P.* *acerifolia* seedlings. After sequencing, 55 known miRNAs and 129 novel miRNAs were obtained, and 104 target genes for the miRNAs were identified by degradome sequencing. Gene Ontology and Kyoto Encyclopedia of Genes and Genomes pathway analyses were performed to predict the functions of the targets. The expressions of eight differentially expressed miRNAs were validated by quantitative real-time polymerase chain reaction (qRT-PCR). This is the first report about *P. acerifolia* miRNAs and their target genes under Pb stress. This study has provided data for further research into molecular mechanisms involved in resistance of *P.* *acerifolia* to Pb stress.

## 1. Introduction

Lead (Pb) is a non-essential element that is toxic to organisms [1]. Pb accumulates in soils via atmospheric deposition. Because of its mobility, Pb is readily taken up by plants where it accumulates in different tissues. Pb inhibits shoot and root growth, reduces photosynthesis, disturbs mitosis and DNA synthesis, and alters enzyme activity [2,3]. It has been reported that excessive generation of reactive oxygen species (ROS) can reduce the speed of DNA replication and inhibit the mitotic process [4,5], which can result in cell membrane peroxidation of lipids and even cause plant death [6]. Scavenging ROS and forming metal chelates are effective mechanisms that plants use for the detoxification of heavy metal stresses [7].

*Platanus acerifolia* is a hybrid of *Platanus orientalis* and *Platanus occidentalis* [8]. It is cultivated widely as a street tree in cities throughout the temperate regions of the world for its good characteristics, such as rapid growth, excellent shadow capability because of its broad crown, root development, and good tolerance to air pollution, that make it amenable to growth in urban environments. The tree has been called the “king of avenue trees” [9]. The use of automobiles is increasingly frequent accompanied by the rapid development of modern social economies and technologies. Pb, a main heavy metal element in automobile exhausts, is constantly released into the urban air. As a result, *P. acerifolia* suffers serious stress from Pb. In recent years, numerous studies on the physiology and biochemistry in *P. acerifolia* have been carried out [10,11], but until now there are no reports about miRNAs in *P. acerifolia* under Pb stress.

MiRNAs are endogenous non-coding RNAs that modulate gene expression either by cleaving target mRNAs to which they bind with near perfect complementarity or by repressing the translation of target mRNAs to which they bind with lower complementarity [12,13]. Double-stranded miRNA/miRNA* is obtained by cutting the stem-loop region of primary miRNAs (pri-miRNAs) [14,15]. The miRNA and miRNA* separate and the miRNA stand is incorporated into the RNA-induced silencing complex (RISC) with the endonuclease Argonaute (Ago) [16]. Several reports have revealed the roles of miRNAs in plant development and hormone signaling [17,18,19,20,21]. Understanding the species-specific roles of miRNAs in response to Pb stress will provide new insights for plant development and for regulating their responses to environmental stresses. To date, the *P. acerifolia* genome sequence has not been published and information about its genetics and functional genes are limited. Deep sequencing of small RNA (sRNA) associated with transcriptome date is an effective way to identify conserved and novel miRNAs. Identifying stress-responsive genes may help provide insights into the complex response mechanisms underlying Pb stress. MiRNAs have been reported to have vital functions in response to a variety of heavy metal stresses in many plants such as cotton [22], *Brassica juncea* [23] and rice [24]. To understand the molecular mechanism of the response of *P. acerifolia* to Pb stress, two sRNA libraries and two degradome libraries were sequenced by high-throughput sequencing, numbers of conserved and novel miRNAs were identified, and the differentially expressed miRNAs and their target genes were analyzed. The results will help lay a theoretical foundation to understand the toxic/tolerant mechanisms of *P. acerifolia* under Pb stress.

## 2. Results and Discussion

### 2.1. Analyses of Small RNAs of P. acerifolia

**Figure 1:**
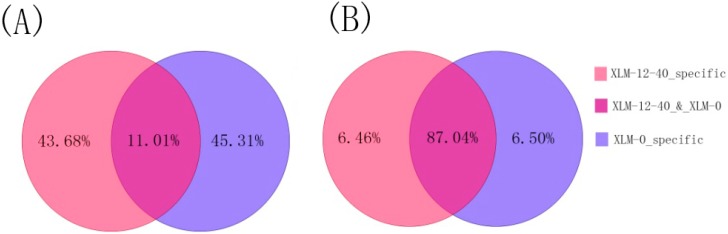
The distribution of sRNA in two libraries (**A**) sRNA counts; (**B**) unique sRNA.

**Figure 2:**
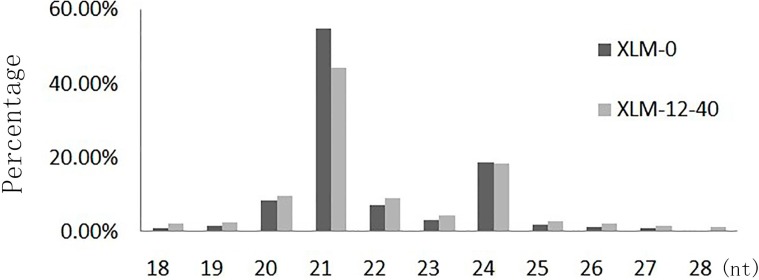
Length distribution of sRNA in two libraries.

Two sRNA libraries were constructed and sequenced by high-throughput sequencing. A total of 14,326,641 raw reads were obtained in the control library (XLM-0) and 14,091,676 raw reads were obtained in the Pb-treated library (XLM-12-40). After removing low-quality reads, adaptors, contaminants, poly(A), and sequences shorter than 18 nucleotides (nt), 13,993,838 (XLM-0) and 13,818,458 (XLM-12-40) clean reads remained. The sequencing statistics for the two libraries are shown in Figure 1. The most abundant sRNA sequences were 21 nt followed by 24 nt (Figure 2) and the percentage of 20–24 nt long sRNAs accounted for 92.94% (XLM-0) and 85.58% (XLM-12-40) of the sequences in the two libraries. The clean reads were mapped to Rfam (http://rfam.sanger.ac.uk/), miRBase, Release 19.0 (http://www.mirbase.org) and the *P. acerifolia* UniGenes, and classified as miRNAs, rRNAs, snRNAs and snoRNAs (Table 1). The proportion of non-annotated unique sRNAs was 93.04% (XLM-0) and 91.50% (XLM-12-40), implying that some unknown sRNAs are yet to be discovered. The non-annotated sRNAs were analyzed to predict novel miRNAs.

**Table 1:** sRNA category and statistics of *P. acerifolia*.

Category	xlm-0				xlm-12-40			
	Unique sRNAs	Percent%	Total	Percent%	Unique	Percent%	Total	Percent%
			sRNAs		sRNAs		sRNAs	
total	1,938,169	100%	13,993,838	100%	1,882,090	100%	13,818,458	100%
miRNA	13,016	0.67%	6,795,537	48.56%	11,264	0.60%	4,945,635	35.79%
rRNA	105,837	5.46%	1,544,175	11.03%	126,468	6.72%	2,464,243	17.83%
snRNA	1206	0.06%	3845	0.03%	1816	0.10%	10,279	0.07%
snoRNA	483	0.02%	1747	0.01%	795	0.04%	3989	0.03%
tRNA	14,316	0.74%	578,095	4.13%	19,698	1.05%	1,226,773	8.88%
unannote	1,803,311	93.04%	5,070,439	36.23%	1,722,049	91.50%	5,167,539	37.40%

### 2.2. Identification of Conserved miRNAs in P. acerifolia

**Figure 3:**
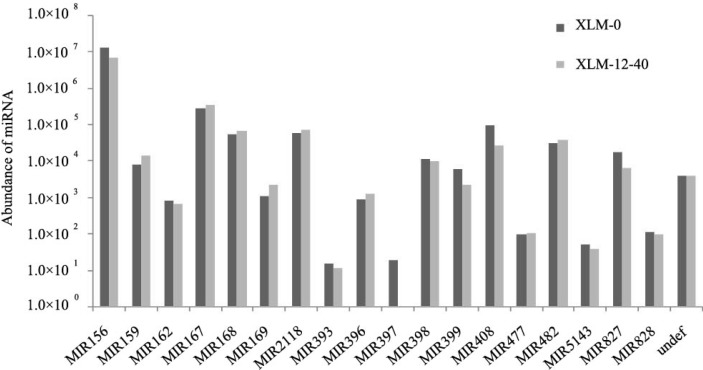
Abundance of conserved miRNAs families in *P. acerifolia*.

To identify the conserved miRNAs of *P. acerifolia*, unique reads were mapped to the mature miRNA sequences in miRBase, Release 19.0, with no more than two mismatches. A total of 55 conserved miRNAs belonging to 18 families were identified, and most of these miRNAs had similar abundances in the two sRNA libraries. MiR156 with eight members was the most abundant family, which accounted for more than 90% of all conserved miRNAs in both libraries (Table S1). The abundances of miR156 family members were between 1384 and 2,484,342 in XLM-0, and between 1135 and 1,300,938 in XLM-12-40; thus, some members of the family had low expression while others had high expression. Notably, the miR156, miR167, miR408, miR2118, miR168, miR482, miR827, and miR398 families were highly abundant in both libraries, while others, such as miR393, miR397, and miR477, had much lower abundances. The abundances of the miRNAs family in the two sRNA libraries are shown in Figure 3. 

### 2.3. Identification of Novel miRNAs in P. acerifolia

To identify potential novel miRNAs, the non-annotated sRNAs were mapped to the *P. acerifolia* UniGenes using SOAP (http://soap.genomics.org.cn/) software. A total of 129 novel miRNAs were identified and 25 of them had the corresponding miRNA*. They included 94 novel miRNAs in XLM-0 and 115 novel miRNAs in XLM-12-40. The minimal folding free energies for the novel precursor pre-miRNA sequences (Table S2) varied from −125 to −19.3 kcal·mol^−1^, with an average of −60.05 kcal·mol^−1^. The novel mature miRNA sequences were 20–23 nt long and 83.72% of them were 21 nt in length. Compared with the conserved miRNAs, the novel miRNAs were lower in abundance.

### 2.4. Analysis of the Differential Expression miRNAs under Pb Stress

To determine the differentially expressed miRNAs under Pb stress, the expressions of all the miRNAs were normalized and their fold-changes and *p*-values were calculated. The expression levels of the miRNAs in the XLM-12-40 library were compared with their expression levels in XLM-0. MiRNAs with *p*-values less than 0.05 and fold-changes >1 or <−1 were designated as “up-regulated” or “down-regulated” in XLM-12-40, respectively. Among the conserved miRNAs, 14 were differentially expressed (one was up-regulated, 13 were down-regulated). Among the novel miRNAs, 67 were differentially expressed (50 were up-regulated, 17 were down-regulated). Among all the differentially expressed miRNAs, pla-miR90, which had the highest fold-change between the two libraries, was down-regulated under Pb stress. The significantly differentially expressed miRNAs between the two libraries are listed in Table S1 and Table S2.

### 2.5. Identification of miRNA Targets by Degradome Sequencing

Most miRNAs are involved in plant resistance to abiotic stress via cleavage of their target genes [25]. To understand the functions and potential regulatory roles of the identified miRNAs, two degradome libraries were constructed as described previously [26,27]. After removing low-quality adapter and redundant sequences, 22,818,398 (XLM-0) and 22,781,045 (XLM-12-40) clean reads were obtained. The CleaveLand pipeline [28] was used to identify the sliced target genes. The miRNA target genes were grouped into five categories (Figure S1) according to the relative abundances of the degradome fragments at the transcript site compared with the abundances of fragments at other sites. A total of 104 targets were predicted to be cleaved by seven conserved miRNA families and 24 novel miRNAs (Table S3) by degradome sequencing. Among these target genes, the largest category in XLM-0 was Category “0” (25 targets), while Category “2” was the largest categories in XLM-12-40 (30 targets). To better understand the roles of the identified miRNAs and illustrate the miRNA-mRNA regulatory network, the target genes were functionally annotated with Gene Ontology (GO) biological process, cellular component, and molecular function terms as described previously [29]. The target genes were classified further using the Kyoto Encyclopedia of Genes and Genomes (KEGG) pathway database (Table S4). The most abundant pathways among the target genes were “Metabolic pathways”, “Biosynthesis of secondary metabolites”, “Plant hormone signal transduction”, “Plant–pathogen interaction”, “Spliceosome”, “RNA transport”, “Endocytosis”, “Glycerophospholipid metabolism”, and “Ribosome”.

### 2.6. Validation of miRNAs and Their Targets with qRT-PCR

To verify the accuracy of the sRNA sequencing results and the potential correlation between the miRNAs and their transcript targets, eight of the differentially expressed miRNAs and eight target genes were selected randomly for validation with qRT-PCR. As shown in Figure S2, the expression patterns of the selected miRNAs determined by qRT-PCR showed similar trends as the corresponding expression patterns in the two libraries as determined by high-throughput sequencing. The results showed that except for the Unigene6816 and CL6054.Contig5 target genes, the expression levels of the other target genes were inversely correlated with their corresponding miRNAs (Figure S3). Interestingly, all the target genes were found to be expressed at a relatively lower level, except for CL11307.Contig2, which was targeted by pla-mir3; pla-mir3 was not expressed in XLM-12-40 through high-throughput sequencing. The expression of pla-mir95 in XLM-0 was seven-fold higher than in XLM-12, while the expression of its target gene, Unigene23581, was almost equal in the two libraries. These results indicated that the expression patterns of miRNAs and their target genes are complex and varied under Pb stress.

### 2.7. Discussion

It has been reported that miRNAs play important roles in the response of plants to environmental stresses [30,31,32]. To understand the roles of *P. acerifolia* miRNAs in regulating gene expression under Pb stress, two sRNA libraries and two degradome libraries were constructed by high-throughput and degradome sequencing, respectively. We found that the most abundant sequences were 21 nt in length (Figure 2), which is consistent with the lengths of the mature miRNAs for eudicotyledons in miRBase (http://www.mirbase.org/cgi-bin/browse.pl), indicating that the two sRNA libraries were likely to contain numerous miRNAs. A total of 55 conserved miRNAs and 128 novel miRNAs were identified in this study. Among the conserved miRNAs, about 61% (33) had more than 100 reads in the two libraries, and only one conserved miRNA had less than 10 reads in the two libraries, indicating that conserved miRNAs were highly expressed. Conversely, only 21% (27) of the novel miRNAs had more than 100 reads in the two libraries, indicating that novel miRNAs were lowly expressed. The numbers of candidate novel miRNAs and their predicted target genes was more than the numbers of conserved miRNAs and their predicted target genes, which is consistent with a previous report that suggested that novel miRNAs may have evolved in plants to help them adapt to different environmental conditions [33].

To help understand the functions of the *P. acerifolia* miRNAs, a total of 124 target genes were identified. Some of the predicted target genes were not annotated in the public databases, suggesting that these unannotated target genes may be in regions of the *P. acerifolia* genome that are not conserved in other plant species [34]. Some of the target genes were transcription factors that may play vital roles in signal transduction under Pb stress [30]. The expression of pla-miR159a-3p was down-regulated in XLM-12-40 compared with its expression in XLM-0, and pla-miR159a-3p was predicted to target a MYB transcription factor. Several reports have found that MYB transcription factors were involved in drought stress [35], salt stress [36], temperature stress [37], metal stress [38], and wounding stress [39]. In common bean (*Phaseolus vulgaris*), MYB transcription factors have been reported to be involved in abscisic acid (ABA) signaling under aluminum stress [40]. Exogenous processing of ABA could significantly increase the activities of superoxide dismutase (SOD), catalase (CAT), ascorbate peroxidase (APX) and glutathione reductase [41]. These enzymes might be regulated by MYB transcription factors through ABA signal transduction. In this study, the expression of a MYB transcription factor (CL13287.Contig1_All) was up-regulated in XLM-12-40, and several MYB transcription factors were targeted by pla-miR159a/b-3p, pla-miR828a/b/c, and pla-mir67a/b-3p, suggesting that these three miRNAs may respond to Pb tolerance by cleaving mRNAs encoding MYB transcription factors.

Immunity in plants might be influenced by abiotic stresses. Their resilience reactions have been considered to be a series of complex signal regulatory network that allow plants to make appropriate response to stresses. In this study, four disease resistance protein RPM1 encoding genes and one nucleotide-binding site (NBS)-containing resistance gene were identified as miRNA targets. It has been reported that most plant disease resistance proteins have supervisory roles in cellular homeostasis [42]. They contain a series of leucine-rich repeats (LRRs), a putative amino-terminal signaling domain, and a NBS, and are termed NBS-LRR proteins [43]. LRR domains have been identified in resistance proteins involved in pathogen recognition and activation of innate immune responses [44]. Further, LRR-receptor-like kinases (LRR-RLKs) were reported to be involved in a diverse signaling processes under biotic and abiotic stresses [45]. In this study, disease resistance protein RPM1 and NBS-containing resistance genes were targeted by the differentially expressed miRNAs pla-miR482a-3p and pla-mir39, implying that a series of signal transduction pathways related to plants’ defense against pathogens might be triggered in *P. acerifolia* under Pb stress. It has been shown that miR169 not only responded to abiotic stresses such as drought and salinity [31,46], but also responded to biotic stresses [47,48]. In this study, pla-miR169a-3p was significantly up-regulated under Pb stress, suggesting that it might be involved in the response to biotic stress in *P. acerifolia*.

Heavy metal stress leads to the accumulation of ROS that can cause damage of lipids, proteins, and nucleic acids and disturb the activity of antioxidative enzymes [49]. Scavenging ROS and forming metal chelates are effective methods of detoxification in plants [7]. ROS accumulation in plants is counteracted by enzymatic antioxidant systems that include scavengers, such as SOD, CAT, and APX [6,50,51]. In this study, several genes encoding proteins involved in scavenging ROS and forming metal chelates were targeted by some of the novel miRNAs. For example, two APX genes that were targeted by pla-mir59 were up-regulated under Pb stress and pla-mir59 was down-regulated, which indicated that APX might be involved in scavenging ROS in *P. acerifolia* under Pb stress. Glutathione S-transferase, which is involved in glutathione metabolism, was reported to not only catalyze the conjugation of glutathione with heavy metal, but also to have peroxidase activity that allowed it to scavenge ROS to relieve oxidative stress [52,53]. Previous study has found that thiol compounds such as glutathione and the phytochelatins were synthesized to reduce free heavy ions in the cytoplasm, which affected the glutathione redox potential [54,55]. Glutaredoxin is an oxidoreductase that can perceive the glutathione redox potential and act as a mediator for the reversible transfer of electrons between the glutathione redox buffer and thiol groups of target proteins [56]. In this study, a glutaredoxin gene was targeted by pla-mir3, and a glutathione S-transferase gene was targeted by pla-mir108-3p and pla-mir45-3p. The qRT-PCR confirmed the expressions of pla-mir108-3p, pla-mir3, and pla-mir59 were down-regulated in XLM-12-40 compared with their expressions in XLM-0, and the expressions of glutathione S-transferase and glutaredoxin were up-regulated in XLM-12-40. These results suggested that there may be synergy among the novel miRNAs pla-mir3, pla-mir59, pla-mir108-3p, and pla-mir45-3p in eliminating ROS and forming phytochelatins in *P. acerifolia* under Pb stress. In addition, analysis of the GO terms assigned to the target genes indicated that they were involved mainly in metabolic processes, and the majority of target genes had functions such as binding, catalytic activity, and transporter activity, suggesting that the target genes might be associated with scavenging ROS and forming phytochelatins.

## 3. Experimental Section

### 3.1. Experimental Materials

Plantlets were cultivated in the Biotechnology Laboratory of Henan Agricultural University, China under an illumination intensity of 130 µmol·m^−2^·s^−1^ and illumination time 16 h·d^−1^ for 60 days at 25 °C. Uniform seedlings were picked out and soaked in 12 g·L^−1^ Pb(NO_3_)_2_ for 40 h for Pb treatment (XLM-12-40) and in distilled water as a control (XLM-0). Each treatment was repeated twice. Five seedlings were selected randomly from each group and 1–4 uppermost leaves of the seedlings were picked separately after 40 h and immediately frozen in liquid nitrogen for RNA extraction.

### 3.2. Construction and Sequencing of P. acerifolia sRNA

Total RNA was extracted using Trizol reagent (Invitrogen, Carlsbad, CA, USA) following the manufacturer’s instructions. Two sRNA libraries (XLM-0 and XLM-12-40) were constructed and sequenced using the Solexa GAIIx platform (Illumina, San Diego, CA, USA). Briefly, the sRNAs were ligated successively with 5' and 3' adapters using T4 RNA ligase (Takara Bio Inc., Dalian, China). Reverse transcription was used to create single-stranded cDNA, which was then amplified by 12 PCR cycles. The cDNA library were purified by polyacrylamide gel electrophoresis (PAGE) to select fragments 140–160 bp in length, which were used to produce the sRNA libraries for high-throughput sequencing. The data used in this publication have been deposited in the NIH Short Read Archive database (http://www.ncbi.nlm.nih.gov/sra) with accession umber SRP056506.

### 3.3. Bioinformatics Analysis sRNA and Identification miRNA of P. acerifolia

After filtering low quality reads, adapters, and contaminated reads from the raw sequence data, clean reads remained and the unique reads among them were selected for analysis. The length distribution of the unique reads was analyzed and the reads were mapped to the *P. acerifolia* UniGenes using miRDeep2 [57]. Perfectly matched reads were analyzed by running Blastall (http://www.ncbi.nlm.nih.gov/staff/tao/URLAPI/blastall/) against the Rfam and GenBank datebases (http://www.ncbi.nlm.nih.gov/) to remove ncRNAs (including tRNAs, rRNAs, snoRNAs, and other ncRNAs). The remaining sRNAs were regarded as candidate miRNAs and were searched against the plant mature miRNAs in the miRBase database (Release 19.0; http://www.mirbase.org/) using Blastall. The reads that aligned to the mature miRNA with a maximum of two mismatches were regarded as conserved miRNAs. To identify novel miRNAs, Mireap (http://sourceforge.net/projects/mireap) and RNAfold (http://rna.tbi.univie.ac.at/cgi-bin/RNAfold.cgi) were used to fold the flanking sequences of the mature miRNAs to predicted secondary structures. The criteria used to select the novel miRNAs were described by Meyers [58].

### 3.4. Analysis of the Differentially Expressed miRNAs under Pb Stress

To identify differentially expressed miRNAs in response to Pb stress, the abundances of the miRNAs in the two libraries were normalized to one million [normalized expression = (number of miRNA reads/total number of clean reads) × 1,000,000]. The normalized reads were used to calculate the miRNA expression levels and *p*-values [59]. MiRNAs with fold-changes >1 or <−1 and *p*-values < 0.05 were considered differentially expressed.

### 3.5. Identification of miRNA Targets with Degradome Sequencing

To predict the potential target mRNAs, two degradome libraries of *P. acerifolia* were constructed as described previously [26,27]. In brief, poly(A) RNA was extracted from the total RNA using a Oligotex mRNA mini kit (Qiagen, Shanghai, China) and an RNA adapter containing a 3' MmeI recognition site was ligated. The ligated products were reverse transcribed and amplified to obtain the PCR products, which were purified and digested with MmeI (New England Biolabs, Ipswich, MA, USA). T4 DNA ligase (New England Biolabs) was used to ligate the digested products, and the PCR products were sequenced on an Illumina HiSeq™ 2000 system (Illumina, San Diego, CA, USA).

After the initial processing, high quality 20–22 nt long sequences were selected for subsequent analysis. The unique sequences were aligned to the database of *P. acerifolia* sequences using SOAP software (http://soap.genomics.org.cn/) to define the coverage rate. Alignments with no more than five mismatches and with no mismatches between the 10th and 11th nucleotides were considered as potential targets. The CleaveLand pipeline [28] was used to find the miRNA targets. According to the abundance of sequences and cleavage sites on the transcript, the cleaved targets were classified into five categories: Category “0”, the most abundant tags are found at the cleavage site and only one maximum on the transcript; Category “1”, more than one tag at the cleavage site, the abundance of tags at the site is equal to the maximum, and there is more than one maximum on the transcript; Category “2”, more than one tag at the cleavage site and the abundance of tags at the site is higher than the median but less than the maximum for the transcript; Category “3”, more than one tag at the cleavage site and the abundance of tags at the site is equal or less than the median for the transcript; and Category “4”, only one tag at the cleavage site. The perfect matched sequences were kept and extended to approximately 35 nt by adding 15 nt of the upstream sequences. All the resulting reads (*t*-signature) were reverse complemented and mapped to the miRNAs identified in this study. *T*-plots were built according to the distribution of the *t*-signatures (and abundances) along the transcripts. The identified targets were subjected to BLASTX analysis and the best hits were used to assign GO annotations (http://www.geneontology.org/) to the targets. Furthermore, to assign pathways, the targets were searched against the KEGG database using Blastall with an *E*-value threshold of <10^−5^.

### 3.6. miRNA Verification by qRT-PCR

Total RNA was extracted using a Plant RNA Extraction Kit (Aidlab Biotechnologies Co., Ltd., Beijing, China) from seedlings soaked in 0, 3, 6, and 12 g·L^−1^ lead nitrate Pb(NO_3_)_2_ for 40 h (XLM-0, XLM-3, XLM-6 and XLM-12, respectively). Three biological replications were run for each sample. qRT-PCR was performed using a SuperScript III Platinum SYBR Green One-step qRT-PCR Fit (Invitrogen, Carlsbad, CA, USA) and CFX96 Real Time PCR system (Bio-Rad, Hercules, CA, USA). The PCR program was run with the following cycles: 50 °C for 3 min; 95 °C for 5 min; then 40 cycles (95 °C for 15 s and 55 °C for 30 s); and 40 °C for 10 min. Technical repetitions were performed three times for each sample. U6 and 18S rRNA were the endogenous internal reference genes for the miRNAs and targets, respectively. The primers used for the qRT-PCR are listed in Table S5. The expression levels were calculated as described by Livak and Schmittgen [60].

## 4. Conclusions

In this study, 55 conserved miRNAs and 129 novel miRNAs were identified, and 104 target genes were detected in *P. acerifolia*. The expressions of some of these miRNAs and their corresponding targets varied under Pb stress, suggesting that Pb could trigger protective mechanisms involving miRNAs that helped enhance the plant’s tolerance to Pb toxicity. These observations contribute valuable information for understanding the gene regulatory frameworks in response to Pb phytotoxicity and provide further insights into the roles of miRNAs and their target genes in regulating *P. acerifolia* tolerance to Pb stress.

## Supplementary Materials

Supplementary materials can be found at http://www.mdpi.com/1422-0067/16/04/7098/s1.
