# Supplementary material for: Identification and Functional Analysis of MicroRNAs and Their Targets in Platanus acerifolia under Lead (Pb) Stress
Source: Int J Mol Sci. 2015 Mar 30;16(4):7098–111. doi: 10.3390/ijms16047098 (PMC4425006; doi:10.3390/ijms16047098)
Supplement: Supplementary file 1 [file ijms-16-07098-s001.zip › ijms-79800-Supplementary Information-Figures.pdf]

Supplementary Information

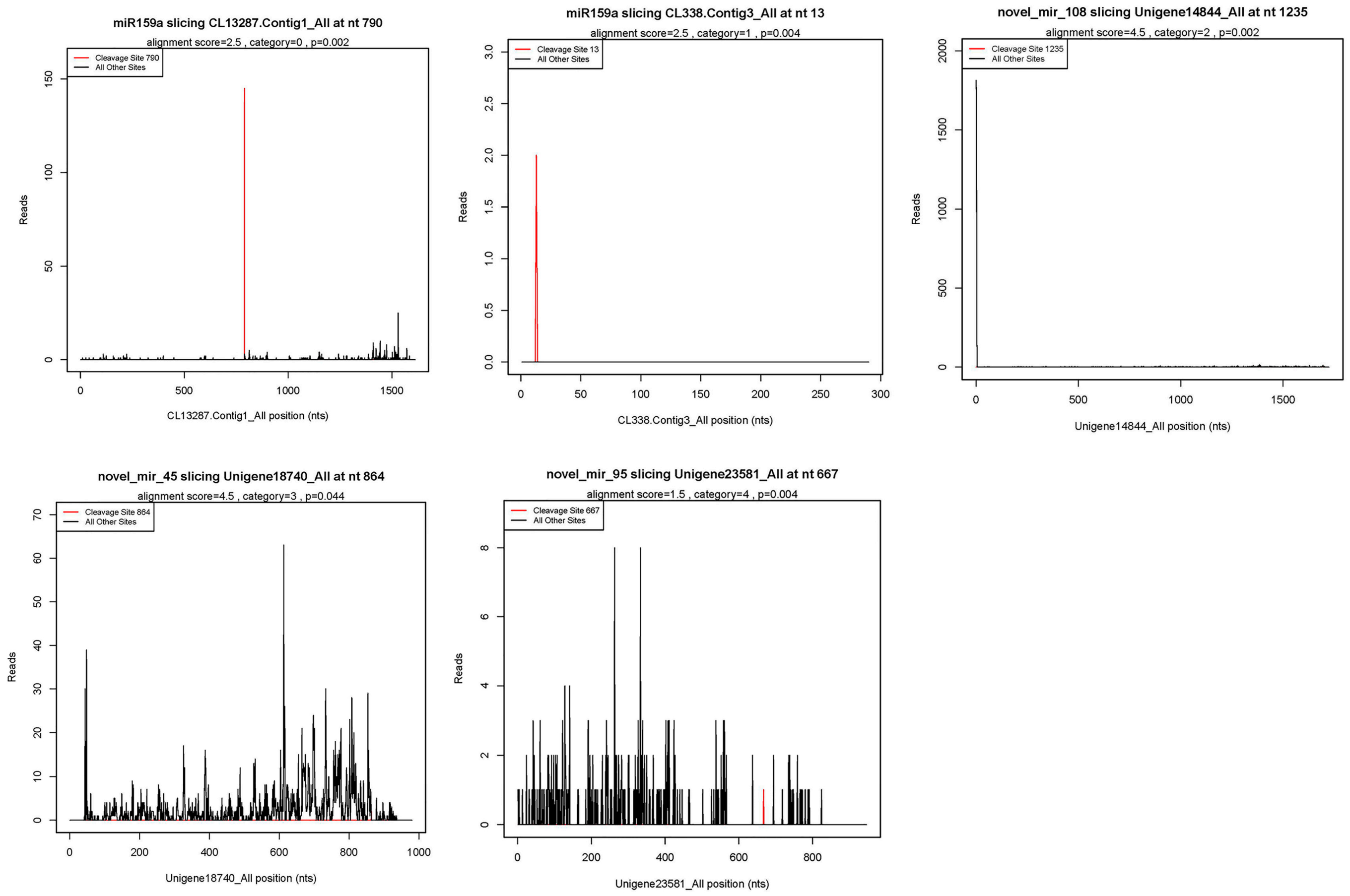

Figure S1. Target plots (*t*-plots) of miRNA targets in different categories confirmed by degradome sequencing.

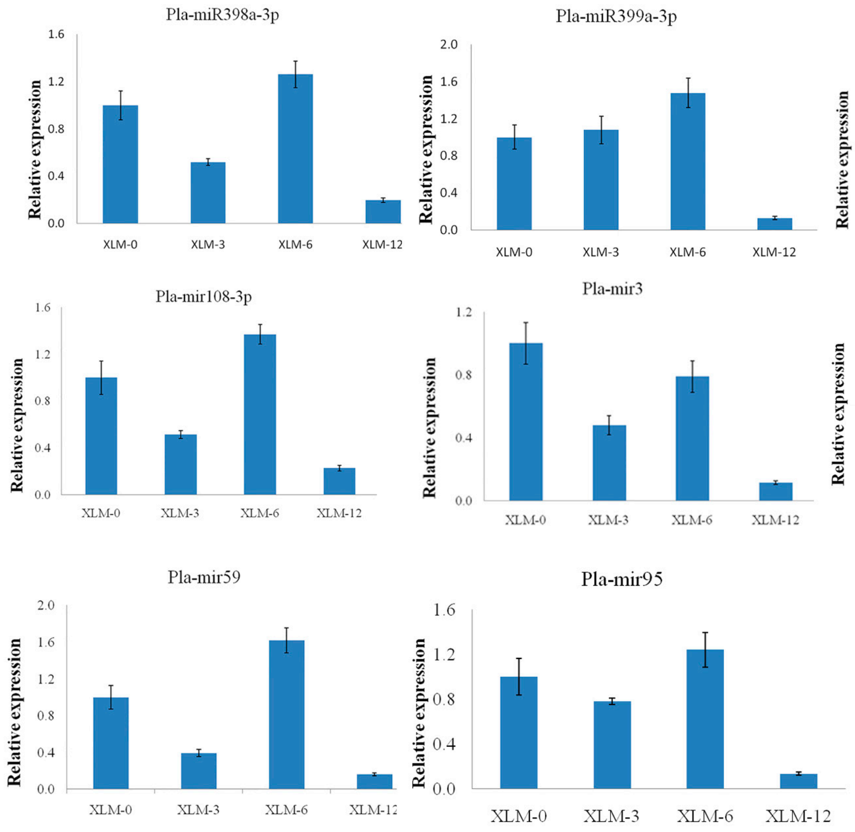

Figure S2. Relative expression levels of the miRNAs in *P. acerifolia*.

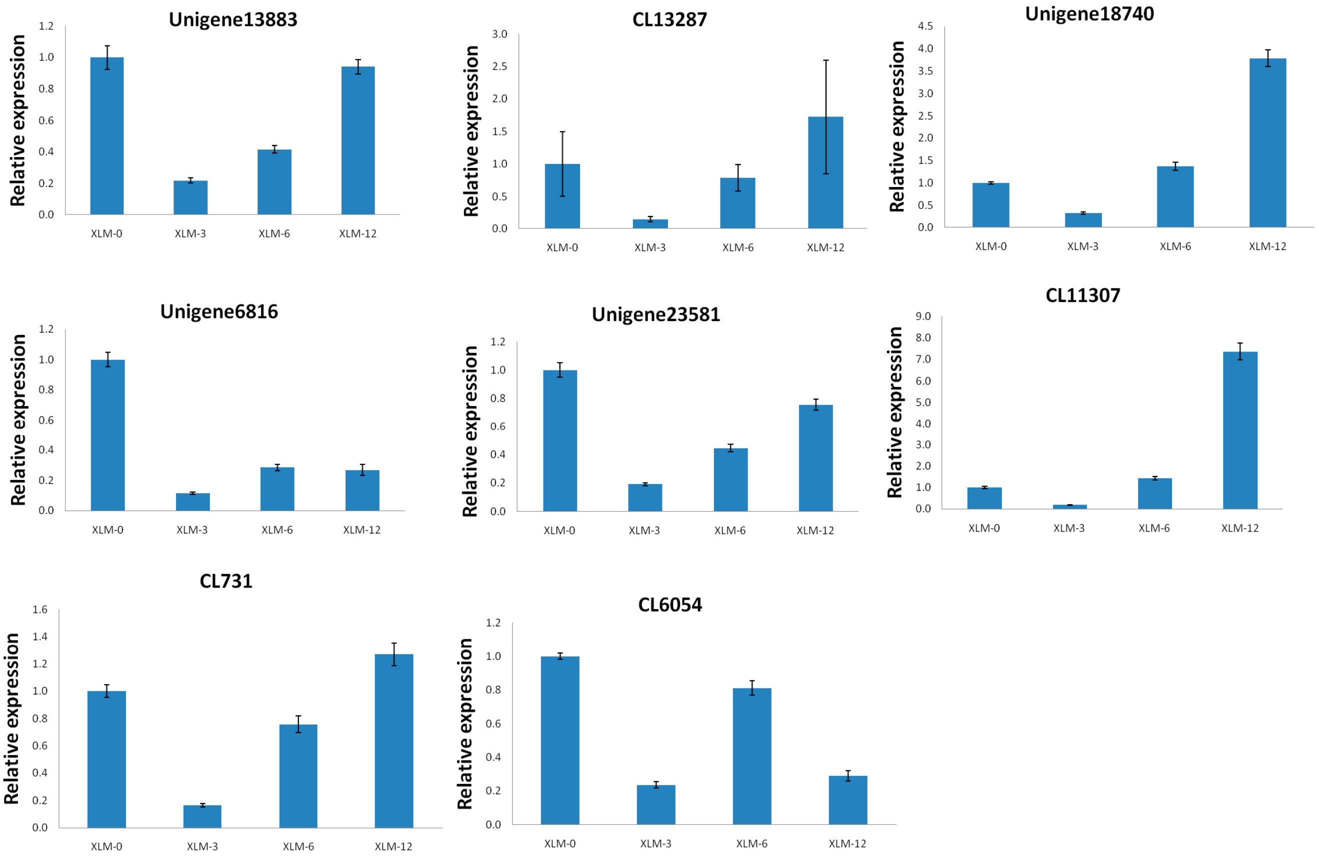

Figure S3. Relative expression levels of the target genes in *P. acerifolia*.
